# 0-Dimensional Persistent Homology Analysis Implementation in Resource-Scarce Embedded Systems

**DOI:** 10.3390/s22103657

**Published:** 2022-05-11

**Authors:** Sérgio Branco, João G. Carvalho, Marco S. Reis, Nuno V. Lopes, Jorge Cabral

**Affiliations:** 1 Algoritmi Center, University of Minho, 4800-058 Guimarães, Portugal; id8676@alunos.uminho.pt (S.B.); b7380@algoritmi.uminho.pt (J.G.C.); 2 CEiiA—Centro de Engenharia, Av. D. Afonso Henriques 1825, 4450-017 Matosinhos, Portugal; 3 DTx—Digital Transformation CoLab, University of Minho, 4800-058 Guimarães, Portugal; nuno.lopes@dtx-colab.pt; 4 CIEPQPF, Department of Chemical Engineering, University of Coimbra, Rua Sílvio Lima, Pólo II—Pinhal de Marrocos, 3030-790 Coimbra, Portugal; marco@eq.uc.pt

**Keywords:** persistent homology, topological data analysis, embedded intelligence, intelligent resource-scarce embedded systems, TinyML

## Abstract

Persistent Homology (PH) analysis is a powerful tool for understanding many relevant topological features from a given dataset. PH allows finding clusters, noise, and relevant connections in the dataset. Therefore, it can provide a better view of the problem and a way of perceiving if a given dataset is equal to another, if a given sample is relevant, and how the samples occupy the feature space. However, PH involves reducing the problem to its simplicial complex space, which is computationally expensive and implementing PH in such Resource-Scarce Embedded Systems (RSES) is an essential add-on for them. However, due to its complexity, implementing PH in such tiny devices is considerably complicated due to the lack of memory and processing power. The following paper shows the implementation of 0-Dimensional Persistent Homology Analysis in a set of well-known RSES, using a technique that reduces the memory footprint and processing power needs of the 0-Dimensional PH algorithm. The results are positive and show that RSES can be equipped with this real-time data analysis tool.

## 1. Introduction

The interest in implementing Machine Learning and Data Analysis algorithms closer to the end-user (edge/end-computing) has increased over the last few years [1,2,3,4]. The need for these algorithms closer to the end-user is due to the need to reduce data traffic and cloud dependency. Therefore, this implementation reduces the device’s power consumption and addresses many security, privacy, and safety concerns. However, the problem with implementing such processing and memory demanding algorithms in Resource-Scarce Embedded Systems (RSES) is the lack of memory, processing capabilities, and arithmetical units. Therefore, to achieve the goal of running these algorithms in RSES, it is vital to re-invent and make optimizations in the algorithms to achieve the same result as in a high-end computer [5].

Persistent Homology (PH) analysis allows understanding of a problem using Topological Data Analysis (TDA). TDA reduces a problem to its topological features, reducing the problem to its simplicial complex space. A simplicial complex space is represented in terms of points, holes, tetrahedrons, and other geometrical shapes. PH takes snapshots of the topological space at each step. This allows finding how the dataset behaves, what can be considered noise during the sampling process to find clusters, and extracting important features. The PH and TDA tools are widely known because Euler used them to solve the Seven Bridges of Königsberg problem. These analysis techniques allow one to reduce the problems into their main features, which turn the increasingly used high-dimensional data into easy-to-see and easy-to-understand problems [6,7].

Persistent Homology analysis has helped solve problems across multiple fields, such as medicine [8,9,10,11], chemistry [12,13], image processing [14], physics [15], astronomy [16], and sensor networks [17,18]. Therefore, Persistent Homology Analysis has been implemented in a wide range of programming languages (e.g., Cpp [19,20,21,22], Python [19,23,24,25,26], Java [27], R [28], Matlab [27,29], and Julia [30]), and it is available in distinct packages that allow using this analysis method across multiple projects. Besides the wide range of software available, none is written in plain C, tested in resource-scarce embedded systems, and neither is designed to perform in such resource-constraint devices.

Besides the many advantages of PH, the tool is computational and memory expensive. The complexity and memory footprint increase as the dimension at which the analysis is made. Therefore, implementing PH in RSES requires looking for novel ways of solving the problem. However, as more and more devices are sampling data in distinct environments, it is necessary to provide RSES with tools for real-time data analysis. This can make the devices understand if the dataset being collected is not behaving as it should or reduce the quantity of traffic sent to the Cloud. Therefore, implementing it into these devices is a challenge but a powerful add-on to reduce cloud dependency and spread the computational work across the network.

The present works focus on the implementation of the data analysis tool, so-called 0 Dimensional PH analysis, proposing a methodology that does not comprise the memory and processing footprint of such resource-constrained devices by resorting to lightweight techniques, such as bitmask and Boolean logic. As shown, the proposed solution can provide outcoming similar PH images but is drawn from fewer samples than the main topological features. With this solution, RSES devices do not need to connect to a Cloud System, which opens the door to making RSES less cloud-dependent and spreading the data analysis task across the network. To the best of our knowledge, this is the first attempt to deploy 0 dimensional PH analysis tool in resource-constrained devices.

## 2. Persistent Homology Analysis

TDA is responsible for extracting the topological features of a given dataset. TDA uses a distance function (e.g., Euclidean Distance). However, instead of just looking at the distance between points, TDA wants to see how the points in a dataset connect. A connection between two points happens if they have a distance equal to or less than *r*. Therefore, one can think of an i-th dimensional sphere around each point with radius *r* and the center equal to the point’s coordinates. If two i-th dimensional spheres touch each other, the points have a connection.

The TDA creates a simplicial space consisting of multiple simplicial complexes. A simplicial complex is a point, a line, a triangle, or any other shape formed by connecting one or multiple points. Therefore, for each possible *r*, there is a single simplicial space. As it is impossible to know which *r* provides the simplicial space that compresses the main topological features, a study is made using multiple *r*s.

PH is the method that takes snapshots of a simplicial space at a given *r* and then stores the information using a barcode. A barcode consists of a bar graph displaying the lifetime of an i-th dimensional hole. Because the main focus of this paper is 0-dimensional PH, one will focus on explaining the behavior of 0-dimensional barcodes and simplicial spaces [31,32,33,34].

In 0-dimensional PH, a hole dies when a connection between two points happens. Therefore, each point in 0-dimensional Homology is seen as a simplicial complex. Therefore, for 0-dimensional PH, one looks at the connection between points and not the empty space between them. One can find possible clusters by keeping track of the number of simplicial complexes at each *r*. Simplicial complexes that die at greater distances are possible point clusters. Simplicial complexes that die earlier are seen many times as noise, and the *r* is used as a filtration value.

For the 0-dimensional PH analysis, it is possible to withdraw two conclusions: The first simplicial complex will die at *r* equal to the minimum distance between points; The last two simplicial complexes join in the worst-case scenario in a *r* equal to the maximum distance between points.

**Figure 1:**
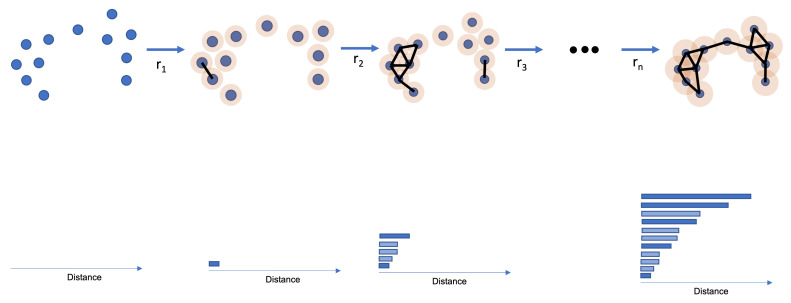
Example of a 0-dimensional Persistent Homology Analysis. At the top, the connections between points form as *r* increases. At the bottom, the barcode originated from the analysis. Each bar represents the distance at which a component has died.

Figure 1 shows how a 0-dimensional PH happens. The top image shows the dataset, with the orange representing the i-th dimensional sphere growing around each point. A connection (black line) appears if two orange circles touch. For each connection, a bar appears at the barcode on the bottom.

## 3. Methodology

The research method followed for the implementation lies in five steps.

The first was to retrieve the barcode image, distance matrix, and simplices information from an existing TDA software. The chosen software was the Ripser (version 0.6.1) from the Scikit-TDA Package. Therefore, the authors verify if the distance matrix obtained from the third-party tool matches the distance matrix built using our method.

The second step focused on building the barcode from the entire dataset using the developed method. Therefore, a comparison with the barcode obtained from Ripser was made. It is verified if the death of the first simplice and the last one occur at the same distance in both tools. Afterward, the authors have withdrawn the unique values from each barcode array, verifying if the values are the same, without a significant gap or a difference higher than 5%. This step verifies that the tool works accordingly to what is expected. Due to the lack of resources in the selected platforms, this step should first be run on a personal computer.

The third step was to verify if the death of the last simplice is the same as the maximum distance found in the distance matrix. Therefore, proving that as soon as one obtains a single simplice, one no longer has to verify for distances higher than the maximum distance already obtained.

The fourth step was to build a visual representation of the dataset using a dimensional reduction tool, verifying if the number of relevant simplices (last three or four) are expected to have larger or smaller values in terms of distance. Therefore, if the given representation provides two clusters significantly distant in the euclidean space, the two bars representing at which distance only two simplices exist up until they merge into one should have a significant difference in size.

The fifth step is to reduce the number of samples through random selection and verify if a smaller batch will provide the same information as the entire dataset. Therefore, proving that the method should and could be implemented into RSES.

The main concern in implementing 0-dimensional homology analysis in a resource-scarce embedded system is the memory needed to hold the entire data structure. Most common implementations of homology use the euclidean distance between points (samples) to analyze, the device needs to store some samples. This set needs to be big enough to make assumptions but small enough not to fill the entire device’s memory. Therefore, the sample matrix and the number of samples (*N*) are the first variables to consider.

Because 0-dimensional homology looks at the distance, the device must also store the distance between samples in a matrix for further analysis. Since the distance matrix has the main diagonal composed of 0 s, and the upper and lower triangles mirror each other, the entire matrix can be compressed into a small array. *N* is the number of samples, and for our method to work, *N* should always be given by $2^{p}$, with *p* an integer higher than one, and p∈N. The array’s size can be withdrawn by re-writing the combination formula as the authors propose in Equation (1).

(1) 2p2=2p!2!(2p−2)!

**Figure 2:**
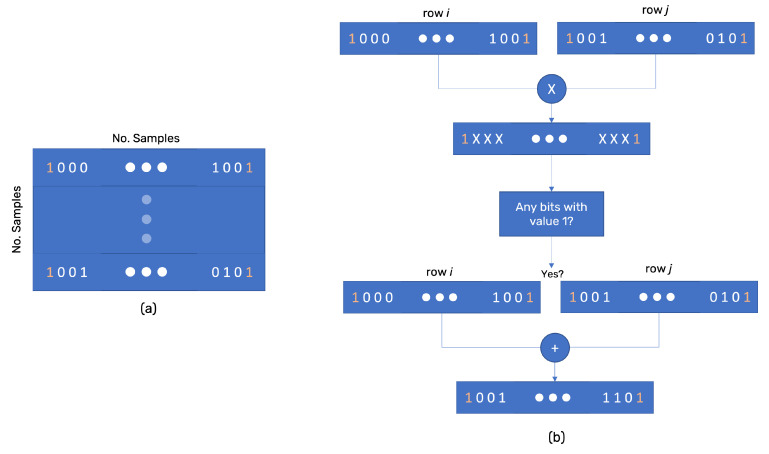
(**a**) The connection matrix has a row size equal to the number of samples. Each row has a bit size equal to the number of samples, implemented using 32-bit unsigned integers. If sample i and j are connected, then bit j in row i has value 1 and bit i in row j has value 1. (**b**) If the bitwise AND result between row i and j have any bit with value 1, the two points have a connection path. If they have a connection path, then the bitwise OR gives us the entire connected points set.

After the distance matrix is calculated, the method iterates through each distance (*r*) inside the distance matrix. The consideration of each *r* being a distance value in the matrix is because the first component will die at the minimum distance existing in the matrix, and two components can only exist until the maximum distance in the matrix. For each *r*, a connection matrix is built. This matrix is a bitmask matrix of row size equal to the number of samples, and each row has a bit size equal to the number of samples (Figure 2a). This is the reason for *N* to be given by $2^{d}$. The bit *j* of row *i* takes the value 1 if sample *i* and *j* are at a distance equal to or smaller than *r*.

The connection matrix allows using bitwise logic to search for connected components. If the AND bitwise logic between rows *i* and *j* have any bit equal to 1, then *i* and *j* are connected. The OR bitwise logic between the two rows (after the AND) gives the entire list of connected points (the simplice). The OR mask is stored at each iteration to avoid repeating simplices, which shortens the processing time. Each simplice is stored and added to a linked list afterward (Figure 2b). Because the matrix has a bit-size of ${\left(N_{samples}\right)}^{2}$, doubling the sampling size quadruples the memory needed. Notice that the connection matrix is a mirror matrix with the main diagonal composed of 1s. Therefore, it is possible to implement the same technique used for the distance matrix. However, it will take more processing but reduce the memory needed.

**Figure 3:**
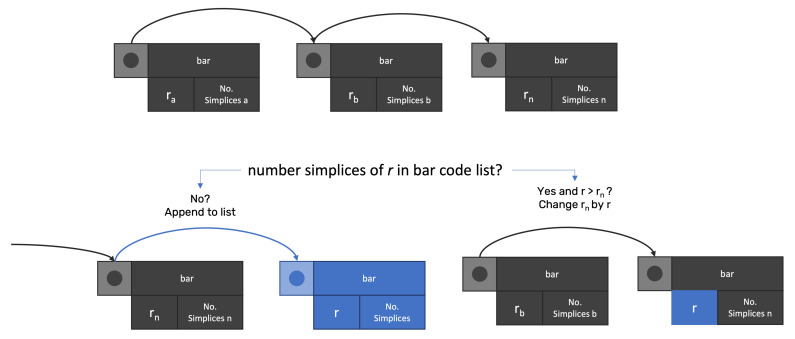
After building the simplices for distance *r*, the method searches any node in the Bar Code list with the same number of simplices. If no node matches the search, a new node is created, storing distance *r* and the number of simplices. If a match happens, it checks if the distance is higher than *r* and changes it if true.

For distance *r*, the method builds the entire set of simplices that exist. Afterward, the method searches in the BarCode linked list for any node with the same number of simplices. If any match occurs, the method verifies if *r* is higher than the distance $r_{m}$ stored in the node. If true, $r_{m}$ is changed by *r*, otherwise, $r_{m}$ continues, as the death value for simplices. If no node matches the simplices’ number, then a new node is created (Figure 3).

Algorithms 1 and 2 explain the pseudo-code used to build the 0-Dimensional Persistent Homology tool.
**Algorithm 1** Build BarCode.build_distance_matrix()**for** r in distance_matrix **do**    **if** r > r_max or r == 0 **then**        ignore    **else**        no_simplices = build_simplices_for_r()    **end if****end for****for** element in simplices_linked_list **do**    **if** element.no_simplices == no_simplices and element.r < r **then**        element.r = r        break    **end if****end for****if** r not in simplices_linked_list **then**    add_to_linked_list()**end if**

**Algorithm 2** Build Simplices for distance r.
**for** i in range(no_samples) **do**    **for** j in range(no_samples) **do**        **if** distance_matrix[i][j] < r **then**           connection_matrix[i] |= (1<<j)        **end if**    **end for**
**end for**
**for** i in range(no_samples) **do**    **if** i not in seen **then**        logical_or_for_all_1_bits()        np_simplices += 1    **end if**
**end for**
return no_simplices


## 4. Experimental Design

The authors have selected a wide range of development platforms that vary in processing power, memory, and hardware to test the hypothesis. The selected platforms and their main specs are present in Table 1. To avoid developing specific code for each platform, the authors have used a generic tool platformio and the same framework (Arduino) to program all boards in equal terms.

The IRIS dataset was selected to check if the results are equal on all platforms and quickly verify the method’s integrity. The dataset is simple in terms of features and classes. However, tests were realized in other datasets to ensure that the results were equally verified.

### 4.1. PCA Representation of the IRIS Dataset

The IRIS dataset represents a classification problem that intends to use four features to classify iris flowers into three species. Therefore, the dataset occupies a four-dimensional Euclidean space. To verify if the results obtained for the 0-dimensional Homology analysis are correct and representative of the dataset behavior, one must try to understand the expected result from such analysis. As the dataset is represented in four dimensions, a Principal Component Analysis (PCA) must be done.

PCA is a tool used to lower the dimension of any dataset. It makes an orthogonal linear reduction that projects the feature space into a subspace generated by a few eigenvectors. So, PCA does not necessarily discard features; it combines them.

**Figure 4:**
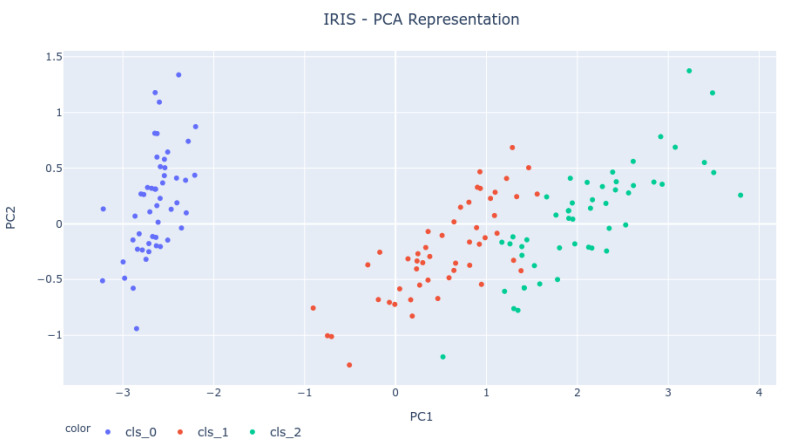
IRIS Principal Components Analysis representation.

Therefore, by making a PCA analysis of the IRIS dataset, it was possible to reduce the dataset into a two-dimensional space (Principal Components 1 and 2), which compresses much of the dataset’s relevant information and allows for a graphical representation of the feature space. Figure 4 shows the PCA representation of the dataset for the first two principal components. As the image shows, the expectation is to see four bars live longer because of the four clusters (three are well visible, and the fourth is composed of the two top-right green dots).

### 4.2. Comparison of Proposed Method against Third-Party Tool Scikit-TDA Risper

**Figure 5:**
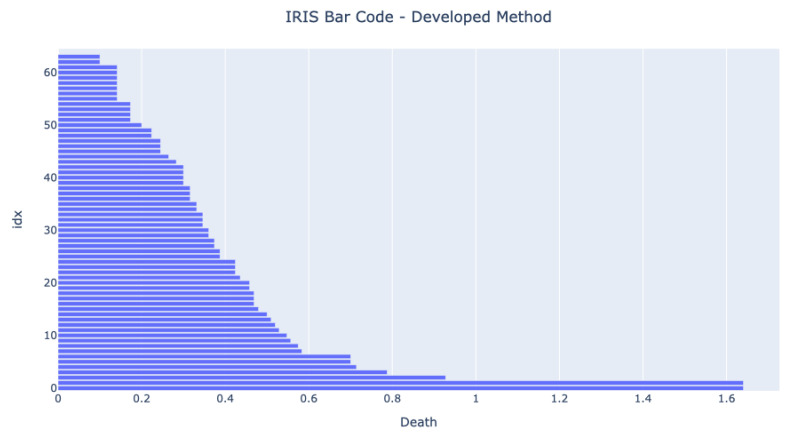
Bar Code obtained from running the developed 0-dimensional homology analysis on the IRIS dataset. The X-axis represents the death of each component. The Y-axis is the array index.

**Figure 6:**
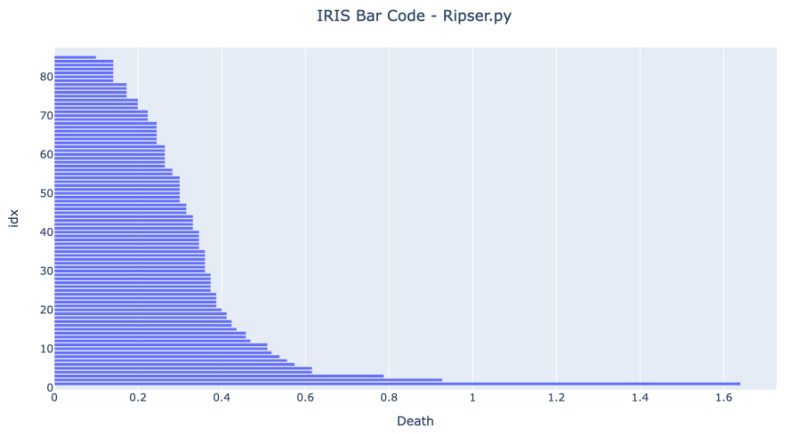
Bar Code obtained from running the scikit-tda ripser 0-dimensional homology analysis on the IRIS dataset. The X-axis represents the death of each component. The Y-axis is the array index.

Before running any tests in an MCU, it was essential to test if the code running in a standard computer could achieve the same results as a third-party generic tool available. The selected third-party tool was the Ripser available in the Python package Scikit-TDA. Figure 5 and Figure 6 show the bar codes obtained by analyzing the dataset in both tools. The results are very similar, and we can see three long bars form. The developed method has one more bar at the end because the authors have decided not to use the infinity value for the one component value but the death value of the second component.

The barcodes are similar, meaning that the developed method provides the same results as other proven methods. The figures show that the expected bar code behavior happens once the three bars that live longer than any other represent the four clusters.

## 5. Results

Table 2 and Table 3 displays the profiling results obtained from running the 0-Dimensional Homology Analysis in each MCU for 32-Samples and 64-Samples, respectively. The 64-Samples analysis was only possible for the STM32F767 because it had enough memory to allocate (100 KB) for the iteration that spent more memory.

As the tables show, every platform could perform the 32-Samples analysis. From the tests, it is possible to understand that an MCU needs to have at least 14KB free for dynamic allocation to perform such analysis. Only the NodeMCU ESP8266 has allocated a different amount of memory, which is not understandable by the bare eye. A possibility is that the NodeMCU implements any memory protection mechanism or alignment to avoid errors or increase performance.

The execution time seems to be mainly linked to the clock speed, but other variables need to be considered. The EK-TM4C123GXL has a slower clock than the Arduino Due, but it runs the analysis slightly faster. One possibility is because the EK-TM4C123GXL has an FPU.

**Figure 7:**
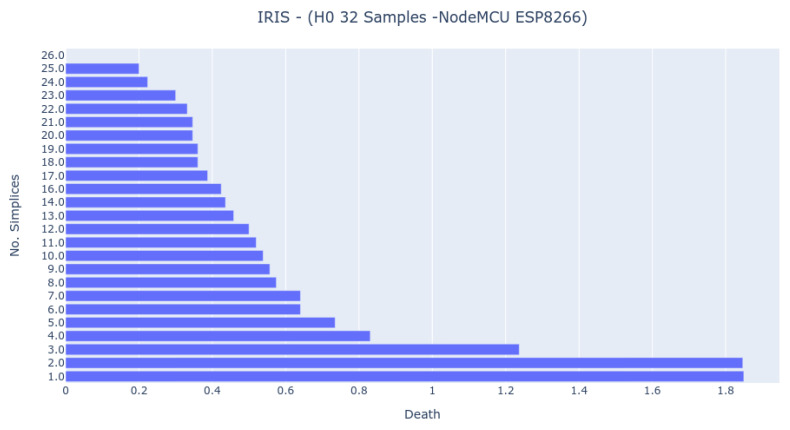
Bar Code result from 0-dimensional Homology analysis in NodeMCU 8266 platform. The analysis was obtained by randomly sampling 32 samples from the IRIS dataset.

The EK-TM4C123GXL has the same clock speed as the NodeMCU ESP8266, but the last one spends less time to achieve a result. However, as Figure 7 shows, the result differs from all the other ones, which indicates that it handles the arithmetics in a distinct way than the others.

**Figure 8:**
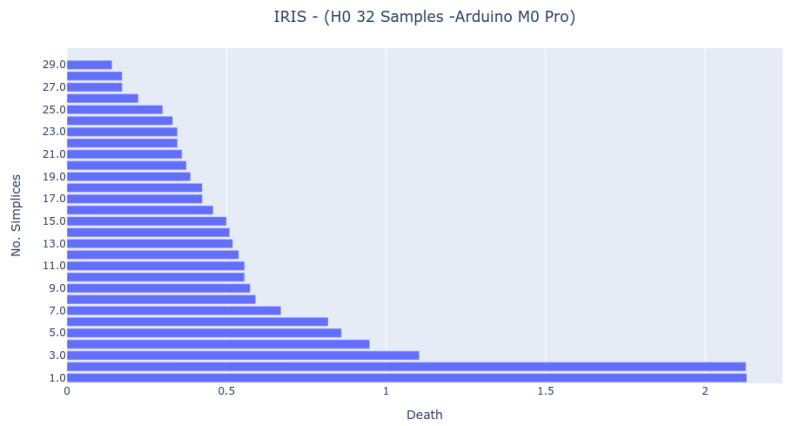
Bar Code result from 0-dimensional Homology analysis in Arduino M0+ Pro platform. The analysis was obtained by randomly sampling 32 samples from the IRIS dataset.

**Figure 9:**
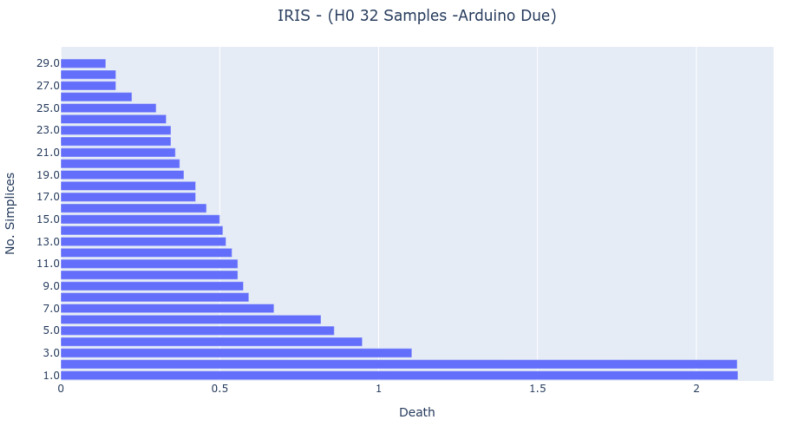
Bar Code result from 0-dimensional Homology analysis in Arduino Due platform. The analysis was obtained by randomly sampling 32 samples from the IRIS dataset.

The Bar Codes in Figure 7, Figure 8 and Figure 9 present the results obtained from running the IRIS dataset 0-dimensional analysis on 32 samples. The barcodes are equal in all platforms (with small differences for the NodeMCU 8266). Therefore, the method is platform-independent. The barcode is also similar to the one obtained from the third-party tool. The important thing to notice is that the death of the last two simplices (to merge into one) happens at a greater distance than all others. This is the expected behavior by looking at the PCA Analysis.

**Figure 10:**
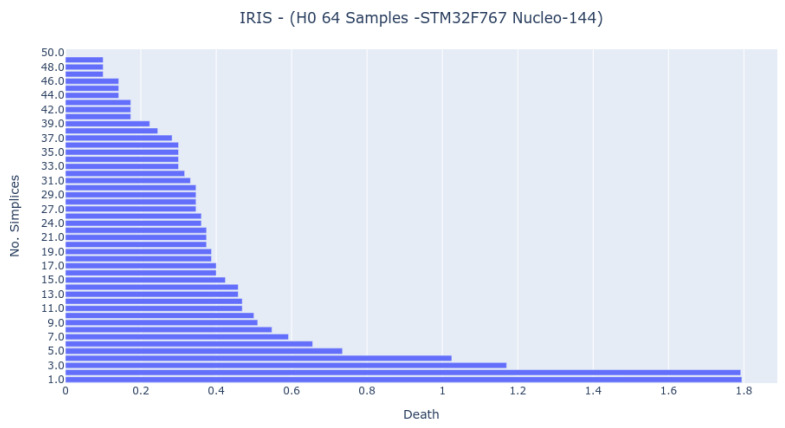
Bar Code result from 0-dimensional Homology analysis in STM32F767 Nucleo-144 platform. The analysis was obtained by randomly sampling 64 samples from the IRIS dataset.

The 64-sample analysis was possible only for the STM32F767 platform (Figure 10). The barcode presents more bars and is closer to the computer analysis, but the most important features exist in all bar codes.

Therefore, the following study shows that reducing the number of samples is possible without losing the persistent homology dataset’s fundamentals. However, this may not be true for all datasets.

## 6. Conclusions

The presented work has shown the implementation of 0-dimensional Persistent Homology analysis in a wide range of Resource-Scarce Embedded Systems. The devices select encompass a variety of MCU architectures, families, and memory/processing capabilities. The work has shown the amount of memory and processing time it takes to achieve the expected result according to the available hardware. Moreover, it is possible to see that the usage of 32/64 samples can achieve the same results as the usage of the entire dataset. Therefore, PH is also a stable and practical method that requires only a variety of samples to present a good image of the entire dataset, at least for a lower-dimensional dataset.

## 7. Future Work

The authors consider that the following future directions of the researcher should be:

- Implementation of higher level PH in RSES for very high dimensional datasets.
- Creation of an algorithm to compare barcodes to verify if the collected dataset represents data obtained previously.
- Develop a dynamic algorithm to re-draw the barcode as new samples arrive.
- Implementation of a federated system to compare multiple barcodes as they are collected.

## Figures and Tables

**Table 1:** Experimental tests platform comparison.

Platform	MCU	Cortex	Clock (MHz)	Flash (kB)	RAM (kB)	FPU
Arduino M0+ Pro	ATSAMD21G18	M0	48	256	32	No
Arduino Due	AT91SAM3X8E	M3	84	512	96	No
EK-TM4C123GXL	TM4C123GH6PMI	M4F	80	256	32	Yes
NodeMCU ESP8266	ESP8266	-	80	1024	128	No
STM32F767ZIT6	STM32F767ZI	M7	216	2048	512	Yes

**Table 2:** Profiling results for 32 samples 0-Dimensional Homology Analysis. The memory is in bytes. The Dynamic Memory field represents the maximum amount of memory used by an iteration. The table is ordered by execution time.

Platform	Flash Memory	Ram Memory	Dynamic Memory	Execution Time (ms)
Arduino M0+ Pro	23,208	7348	13,828	959
Arduino Due	18,396	6764	13,828	505
EK-TM4C123GXL	14,797	8293	13,828	422
NodeMCU ESP8266	270,101	33,188	15,856	267
STM32F767ZIT6	21,596	6292	13,828	70

**Table 3:** Profiling results for 64 samples 0-Dimensional Homology Analysis. The memory is in bytes. The Dynamic Memory field represents the maximum amount of memory used by an iteration. The table is ordered by execution time.

Platform	Flash Memory	Ram Memory	Dynamic Memory	Execution Time (ms)
STM32F767ZIT6	23,070	19,399	98,344	1055

## Data Availability

The complete code implementation is available at https://github.com/asergiobranco/mcu_homology (accessed on 15 January 2022).
